# Perception of Apps for Mental Health Assessment With Recommendations for Future Design: United Kingdom Semistructured Interview Study

**DOI:** 10.2196/48881

**Published:** 2024-02-23

**Authors:** Erin L Funnell, Benedetta Spadaro, Nayra A Martin-Key, Jiri Benacek, Sabine Bahn

**Affiliations:** 1 Cambridge Centre for Neuropsychiatric Research Department of Chemical Engineering University of Cambridge Cambridge United Kingdom; 2 Psyomics Ltd Cambridge United Kingdom

**Keywords:** app design, digital health, eHealth, interviews, mental health, mHealth, mobile phone

## Abstract

**Background:**

Mental health care provision in the United Kingdom is overwhelmed by a high demand for services. There are high rates of under-, over-, and misdiagnosis of common mental health disorders in primary care and delays in accessing secondary care. This negatively affects patient functioning and outcomes. Digital tools may offer a time-efficient avenue for the remote assessment and triage of mental health disorders that can be integrated directly into existing care pathways to support clinicians. However, despite the potential of digital tools in the field of mental health, there remain gaps in our understanding of how the intended user base, people with lived experiences of mental health concerns, perceive these technologies.

**Objective:**

This study explores the perspectives and attitudes of individuals with lived experiences of mental health concerns on mental health apps that are designed to support self-assessment and triage.

**Methods:**

A semistructured interview approach was used to explore the perspectives of the interviewees using 5 open-ended questions. Interviews were transcribed verbatim from audio data recordings. The average interview lasted 46 minutes (rounded to the nearest min; SD 12.93 min). A thematic analysis was conducted.

**Results:**

Overall, 16 individuals were interviewed in this study. The average age was 42.25 (SD 15.18) years, half of the interviewees identified as women (8/16, 50%), and all were White (16/16, 100%). The thematic analysis revealed six major themes: (1) availability and accessibility, (2) quality, (3) attitudes, (4) safety, (5) impact, and (6) functionality.

**Conclusions:**

Engaging in clear communication regarding data security and privacy policies, adopting a consent-driven approach to data sharing, and identifying gaps in the app marketplace to foster the inclusion of a range of mental health conditions and avoid oversaturation of apps for common mental health disorders (eg, depression and anxiety) were identified as priorities from interviewees’ comments. Furthermore, reputation was identified as a driver of uptake and engagement, with endorsement from a respected source (ie, health care provider, academic institution) or direct recommendation from a trusted health care professional associated with increased interest and trust. Furthermore, there was an interest in the role that co-designed digital self-assessments could play in existing care pathways, particularly in terms of facilitating informed discussions with health care professionals during appointments and by signposting individuals to the most appropriate services. In addition, interviewees discussed the potential of mental health apps to provide waiting list support to individuals awaiting treatment by providing personalized psychoeducation, self-help tips, and sources of help. However, concerns regarding the quality of care being affected because of digital delivery have been reported; therefore, frequent monitoring of patient acceptability and care outcomes is warranted. In addition, communicating the rationale and benefits of digitizing services will likely be important for securing interest and uptake from health care service users.

## Introduction

The demand for mental health care is steadily rising [1], with mental health disorders being among the leading causes of global disease [2] and disability burden [3]. Despite the documented consequences of delays in providing support [4,5], there is inadequate service coverage and poor quality of care within most health care systems globally [6], with documented disparities between the quality of physical and mental health care [7].

In this regard, mobile and internet technologies, such as web and smartphone apps, have been identified as potential tools to improve access to care by mitigating some of the economic, geographic, and human resource constraints posed by in-person care [8]. Evidence gathered from numerous studies supports the view that the internet and mobile apps have become a source of health information, support, screening, and disease management, as well as being the entry point for referral processes with various degrees of success in implementation.

In the United Kingdom, there is an urgent need for innovative mental health technologies, as despite a lifetime prevalence of 1 in 4 adults experiencing a mental health disorder [9], many individuals who seek help face considerable barriers to effective support. First, an estimated 1 in 35 people are waiting for specialist mental health care in the United Kingdom [10], with the reported wait for specialist care often exceeding a month [4]. Patients who spend more time on waiting lists report worsening mental health symptoms [4,5]. In addition, there are reports of impairments in social or relationship domains, with some individuals reporting that their mental health deteriorated to the extent that they needed emergency care [4].

Second, there is well-documented evidence of mental health misdiagnosis. It is estimated that 90% of mental health concerns are managed entirely in primary care settings [11]. Despite this, there is frequent misidentification of common mental health disorders, such as major depressive disorder [12,13], bipolar disorder [14], and anxiety disorders [15]. There is also evidence that some general practitioners (GPs) feel their surgeries are underprepared to provide adequate mental health care [16]. A plethora of interconnected reasons may explain the misdiagnosis of mental health conditions, such as the overlapping symptom profiles of different psychiatric disorders [17], short consultations [18], and a lack of appropriate training [19]. These assessment difficulties are compounded by person-level barriers, such as difficulties in accurately communicating symptoms to health care professionals [20]. Additional barriers to diagnosis include geographic variability in resources and the availability of trained mental health care professionals.

In contrast, smartphone ownership is becoming widespread, all-time high, and the increasing number of mobile device users has created an unprecedented opportunity to develop evidence-based mobile apps for remote delivery of mental health care. There is evidence of interest from individuals with mental health concerns in digital tools designed for mental health assessments, particularly when they are integrated into care pathways, with results delivered directly to a health care professional before their appointment [5]. Individuals appear to feel more comfortable disclosing sensitive information digitally [21] than health care professionals. In addition, digital self-assessment and triage support tools may be useful in augmenting existing services by ensuring timely response and intervention to urgent cases [22,23] and signposting appropriate services outside formal health care.

However, despite the interest in and promise of mental health apps, prolonged engagement and use remains an issue [24]. This poses the question of how to design tools that are usable and useful for individuals experiencing mental health concerns. Iterative user-centric research and design are key to addressing this challenge. Indeed, work has been done to explore the opinions of apps designed for mental health concerns and disorders [25-29]. However, given the interest in digital tools intended for mental health self-assessment and triage, more work is required to understand the perspectives and acceptability of such tools designed and implemented to augment existing care pathways.

Therefore, in this study, we conducted semistructured interviews with individuals who represented potential app users: those with lived experiences of mental health concerns. The key objective of this study is to advance the understanding of potential users’ views and perspectives on mental health apps, with a specific focus on apps designed for self-assessment and triage. To this end, the semistructured interview included eight questions broadly focused on the following topics: (1) previous use and perception of mental health apps; (2) perspectives of mental health assessment apps; (3) perspectives of opportunities for integration of mental health apps, including mental health assessment apps, into traditional care; (4) perspectives of the safety and privacy aspects of mental health apps; and (5) desired app features for an ideal mental health app.

## Methods

### Overview

The methods and results presented in this study are reported in-line with COREQ (Consolidated Criteria for Reporting Qualitative Studies [30]; Multimedia Appendix 1). The research team comprised 3 research assistants (ELF, BS, and JB), a research associate (NAM-K), and a practicing psychiatrist and professor of neurotechnology (SB). All authors had previous experience in qualitative data analysis. JB is identified as male, and all other authors are identified as female. This study was a follow-up to a previous web-based survey study [5].

### Participants and Recruitment

Participants from a previous web-based survey study [5] who expressed interest in participating in follow-up studies were contacted via email between April and June 2022 and invited to participate in this interview study. The email addresses collected in the prior survey study [5] used to contact potential participants for this study were stored in a locked (password-protected) Excel file, only accessible to members of the research team who were named after ethical approval. The inclusion criteria were as follows: (1) aged ≥18 years, (2) living in the United Kingdom, and (3) having to visit a health care professional after 2016 to discuss their mental health symptoms. The first 2 rounds of recruitment were blinded to the demographic characteristics. In the second and third rounds of recruitment, only participants who had identified as men or nonbinary in the survey study were contacted to increase the representation of these groups in this study sample.

### Study Procedures and Materials

Participants were first provided with written details of the aims, methods, and requirements of this interview study via email. They were then asked to express their interests and provide consent to participate via email. Upon the expression of interest and receipt of consent, the date and time for the interview agreed with the researchers (ELF and BS). The interviews were conducted by an interviewer (ELF or BS) in the presence of an observer (ELF or BS).

All study materials, including the participant information sheet, consent form, interview guide, and debriefing, were developed in consultation with the senior author (SB) of a practicing psychiatrist. In addition, some questions in the interview guide were adapted from previous relevant literature investigating attitudes toward digital interventions for mental health [31,32]. All study materials were then further amended and finalized in consultation with members of the Cambridge University Hospitals Patient and Public Involvement panel, who have lived experiences of mental health concerns. This strategy was used to ensure the suitability and relevance of the study materials to the target population.

A semistructured interview guide was used to facilitate the conversation, which included open-ended questions to encourage participants to discuss their perceptions of digital tools (eg, applications) for mental health. The guide included eight questions (with further prompts) focusing on (1) previous use of mental health apps, (2) perceptions of mental health apps, (3) perspectives of apps for mental health assessment, (3) perspectives of receiving an indication of a mental health diagnosis from an app, (4) perspectives of information collected by a mental health assessment app being sent to a health care professional, (5) perspectives on whether apps can improve access to mental health services, (6) views on safety and privacy related to mental health apps, and (7) desired features for future mental health apps. In addition, the participants were asked (8) if they had any additional thoughts relevant to mental health and digital technologies (eg, apps). A copy of the semistructured interview script is available in Multimedia Appendix 2. The interviews were adaptive, such that only relevant questions were asked based on previous responses. The interviewer could reformulate or clarify questions during the interviews to gain a deeper understanding of the participants’ thoughts and opinions, or delve into relevant details that were mentioned in relation to the questions asked. The audio of the interview was recorded for subsequent transcription. After completion of the interview, all participants were provided with debrief via email and offered a £15 (~US $18) Highstreet voucher for their time.

### Data Collection

Interviews were conducted between May 5, 2022, and June 22, 2022. Overall, 14 (88%) of the interviews were conducted using Zoom (Zoom Video Communications Inc) videoconferencing, 1 (6%) using Microsoft Teams videoconferencing, and 1 (6%) via telephone call. Participants were informed that they did not need to have their cameras on during the interview. Audio data were recorded for all the interviews. In total, 12 hours and 25 minutes (rounded to the nearest min) of interview audio were recorded, with the average length of the interview being 46 minutes (rounded to the nearest min; SD 12.93 min). The interviews were transcribed verbatim from audio data recordings using cloud-based AI-powered software Otter [33]. The researchers (ELF and BS) then reviewed the transcripts by listening to the audio recordings and amending the transcripts where necessary. Any unclear audio segments were labeled “unintelligible.” Interview transcripts were numbered and not connected to any identifiable information (ie, email address for recruitment and participation reimbursement). Any names or identifying information (eg, city of residence) disclosed during the interviews were removed from the transcripts. The transcripts were downloaded as PDFs and analyzed as described below.

### Data Analysis

The data were analyzed using a bottom-up (data-driven) thematic analysis approach based on the Braun and Clarke framework [34]. A total of 2 authors (ELF and JB) analyzed all the transcripts under blinded conditions using the following process: the first interview transcription was analyzed, and initial codes were identified. The second interview was analyzed by checking for the presence of codes identified from the analysis of the first interview and adding any new codes identified. This process was continued for each interview transcript, each time adding or refining existing codes. Upon completion of the analysis, the authors were unblinded and compared their lists of identified codes. Any inconsistencies were discussed with a third author (BS or NAM-K) until consensus was reached. Once the codes were agreed upon by all authors, they were organized into themes under blinded conditions by 2 authors (ELF and JB). The resulting themes were discussed with a third author (BS and NAM-K) until a consensus was reached. Data analysis (ie, code creation and assignment, theme creation, and assignment) was performed using Google Sheets spreadsheets. A copy of the codebook organized into their respective themes is available in Multimedia Appendix 3.

### Ethical Considerations

This study was approved by the University of Cambridge Human Biology Research Ethics Committee (approval number: PRE.2021.053). The participants provided informed consent electronically via email to participate in the study. In one case, a participant provided consent at the start of the interview because they had experienced difficulties in sending a complete consent form via email. On the day of the interview, the interviewer (ELF or BS) verified that participants understood the information that had been provided to them and gained verbal consent that they were happy to continue with the interview.

## Results

### Sociodemographic Characteristics

A total of 16 individuals were included in this study. The average age was 42.25 (SD 15.80), with 50% (n=8) of the participants identifying as women and all White (16/16, 100%). English was the native language of 87% (14/16) of the interviewees. More than 69% (n=11) had at least one undergraduate degree. A total of 43% (7/16) were single and 43% (7/16) were cohabiting. Regarding accommodation characteristics, living alone or with a partner was the most common arrangement, with 38% (n=6) living alone, 25% (n=4) living with a partner, and 19% (n=3) living with a partner or children. A total of 37% (6/16) were employed, and 62% (10/16) had a household income of less than £35,001 (approximately US $43,608) before tax.

### Mental Health Characteristics

The majority (15/16, 94%) of respondents had discussed their mental health with a GP in the last 5 years, with 88% (n=14) having also seen a therapist or counselor. More than half (9/16, 56%) of the participants had also seen a psychiatrist. Mental health care visits were typically provided free of charge via the National Health Service (15/16, 94%). A total of 81% (13/16) of the interviewees were diagnosed with a mental health disorder (see Table 1 for a breakdown of the frequency of diagnoses). The most common diagnosis in the sample was major depressive disorder (13/16, 81%), followed by generalized anxiety disorder (5/16, 31%), and posttraumatic stress disorder (5/16, 31%).

**Table 1:** Frequency of mental health diagnoses in the sample (N=16).

Mental health condition	Frequency, n (%)
Major depressive disorder	13 (81.25)
Bipolar disorder	2 (12.50)
Generalized anxiety disorder	5 (31.25)
Social anxiety disorder	3 (18.75)
Panic disorder	1 (6.25)
Obsessive-compulsive disorder	1 (6.25)
Sleep disorder	2 (12.50)
Posttraumatic stress disorder	5 (31.25)
Personality disorder	2 (12.50)
Eating disorder	2 (12.50)
Attention-deficit/hyperactivity disorder	3 (18.75)
Autism spectrum disorder	1 (6.25)
Learning disability	1 (6.25)

### Thematic Analysis

**Figure 1:**
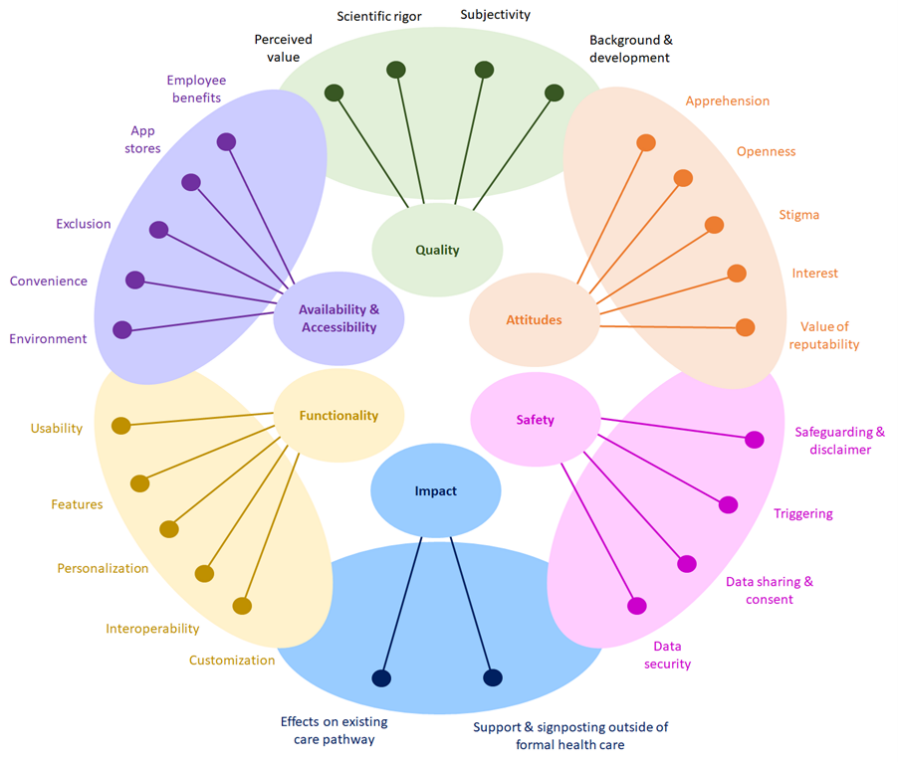
The 6 major themes and corresponding minor themes that emerged from the data (illustration inspired by de Angel et al [35]).

Thematic analysis revealed 6 major themes comprising 25 minor themes (Figure 1).

#### Availability and Accessibility

Availability and accessibility emerged as key themes in this study, with issues surrounding (1) app stores, (2) exclusion, (3) environment, (4) convenience, and (5) employee benefits emerging as important minor themes.

Regarding the first minor theme, app stores, interviewees reported that there is too much variability in what app store searches return, which could make app stores difficult to navigate and, in turn, be overwhelming for users:

It’s overwhelming and it can be just really difficult to find what you’re looking for as well. You know, if you just put in a search term, and then you just get kind of a load of random stuff. That’s not necessarily categorized very well, I don’t think.

Transcript 5

In addition, concerns were raised about groups of individuals with specific conditions being excluded from the adoption of a digital approach to mental health, with gaps in the market for apps for certain mental health conditions, such as neurodevelopmental disorders:

[...] specialized things like ASD specifically, I’ve not really seen anything in terms of like apps to help with anything like that sort of helping with meltdowns and things like that.

Transcript 4

Interviewees also noted a lack of diversification in-app offerings, expressing concerns that available apps are basic and essentially all offer the same thing:

I think the ones that the NHS or the GP or the CPN have recommended they seem to be quite basic. I supposed cause the NHS makes them, they make it for everybody, so you can’t really fine tune it, I don’t think.

Transcript 15

But in terms of my perception, I think, I don’t think they have a lot of options out there. I mean, there’s a broad range. I mean, you’ve got apps, that you know, under different names, but they all, they all work the same.

Transcript 13

In terms of the second minor theme, exclusion, interviewees raised concerns related to severe or complex mental health symptoms impeding users’ ability to actively engage with a mental health app:

I don’t think I’d have the mental abilities to read a question, understand it, put in an answer, look at my answers, look for any pattern there might be or anything like that. I think I wouldn’t be able to do it.

Transcript 6

In addition, with regard to exclusion, not being able to afford a potential one-off or subscription payment was raised as a concern:

I know some of them have like paid options, which sometimes can be a bit of a barrier. I’m in a position now where I can like pay for subscriptions, but in the past like when I was a university student I think that was one thing that sometimes put me off was having to pay for them. I used to use one calm. I found it was actually quite expensive and there’s like other apps that are completely free, that do the same thing.

Transcript 7

Moreover, exclusion due to digital delivery, particularly in the elderly, those with limited digital literacy, or individuals with physical health conditions such as visual impairments or dyslexia, was raised as an important barrier:

[...] it would exclude some people who haven’t got the internet. And it would exclude a lot of older people who might have it and haven’t got a clue how to do it. So, so that would be one of the disadvantages of excluding some people [...].

Transcript 6

The environment was also identified as a minor theme within the availability and accessibility theme, with interviewees raising the issue of not having the right environment to complete a digital mental health assessment:

[...] when you're in a house when everybody else is stuck at home as well you don’t get peace and quiet. You actually need to concentrate on it. [...]. When you’re trying to sit down at home in a busy house doing it, you’re never guaranteed to have the time you need to actually finish the section in one go, having to restart things three sometimes four times just to actually work through it.

Transcript 8

In contrast, interviewees also reported that digital technologies are convenient and improve accessibility to mental health services:

[...] a lot of people can’t, can’t get to places. I don’t just mean COVID I mean, they haven’t got the money or it takes longer or whatever.” “But then the same things that are advantages are disadvantages. Because like people who are physically disabled, it must, it’ll be a lot easier for them to use to do things online.

Transcript 6

as well as fitting well into modern daily routines:

I find that the good thing of them, is that you can use them, do them anytime. Can do it at your own pace and at home so you don’t have to depend on anyone to do it or, or have a scheduled time to.

Transcript 14

Finally, regarding the role of the workplace, it was noted that engagement with mental health apps could improve if provided as an employee benefit:

Yeah, I mean, I got it free from my work. They had an agreement with them, so I feel I’ll try it anyway.

Transcript 11

#### Quality

The quality theme was identified in this study, encompassing the minor themes of (1) subjectivity, (2) scientific rigor, (3) background and development, and (4) perceived value.

Interviewees mentioned the subjective nature of mental health symptoms. Because of their reported subjective nature, symptoms were deemed difficult to consolidate with the formal diagnostic labels and descriptions of mental health disorders, causing distrust regarding the validity of diagnoses:

I must say it’s all very subjective, isn’t it? Your own feelings are all what you feel and then you think just that is really what they mean? Am I feeling what they, what they mean?

Transcript 3

Related to this aspect of subjectivity, interviewees mentioned concerns related to self-reporting of mental health symptoms in self-assessments. Some interviewees expressed that the subjective and hard-to-define nature of mental health symptoms can create a risk that users of mental health apps may unintentionally report their symptoms inaccurately:

So[...] you know the thing is people are expressing their symptoms, but they might be inaccurate symptoms. It might only be that person’s interpretation of what they’ve got.

Transcript 3

Others have stated that app users may intentionally incorrectly report or exaggerate their symptoms to support self-diagnosis of a specific mental health disorder:

I guess there’s always the risk of people not being honest. And maybe people exaggerating symptoms or something like that. I know there’s a lot of talk at the minute, isn’t it about social media and people like self-diagnosing from social media, and I know it’s only a small percentage of people, but I guess that would be a concern.

Transcript 7

The subjective nature of mental health symptoms and concerns related to self-reporting are both associated with potential difficulties in using digital tools for accurate mental health assessment, with interviewees reporting concerns that mental health symptoms may be difficult to capture in assessments that rely on predefined multiple-choice questions:

[...]it’s difficult if you’re, what you’re experiencing doesn’t fit in with the responses that have been configured.

Transcript 1

I’d be concerned about using the app on its own to do a mental health assessment just because I think it can lack nuance sometimes.

Transcript 16

One potential method, offered by an interviewee, to address the challenges of subjectivity outlined here was to allow for clinician-facilitated symptom reporting, wherein the app user and clinician could collaboratively complete the mental health assessment. This collaboration would allow the user to provide details of their symptoms or experiences, which the clinician can use as a basis to answer the questions within the assessment:

[...]the GP, for example, could go up to[...] his or her clients and say, ‘Oh, what do you want me to put to this question’ and that, they’ll just give you, the client will just give you a tidbit and then they’ll just put that down. I think that would help.

Transcript 13

In terms of scientific rigor, interviewees expressed concerns about a mental health assessment app’s diagnostic accuracy, stating that apps may be able to suggest a condition that is a probable explanation for a user’s mental health symptoms but that it would likely not be completely accurate:

I don’t think an app will ever truly get a diagnosis correct. It’ll probably get a rough estimate.

Transcript 13

Interviewees also raised concerns that due to the overlapping symptom profiles of many mental health disorders, there is a potential risk of misclassification of symptoms:

I think not too sure about the apps for online diagnoses in general as well. I suppose with a lot of the conditions, the elements of different mental health issues, it might not be that you’ve got say bipolar [...] you might have a different mental health condition and you seem to fit the criteria for bipolar.

Transcript 15

With these concerns about diagnostic accuracy, interviewees reported a preference for apps that provide mental health screening to indicate probable present conditions, which can then be confirmed by a mental health care professional, over formal mental health diagnosis:

I don’t think a doctor ought to diagnose someone on the basis of an app. I think they both have to go together.

Transcript 6

In addition, a preference for an indication of where their symptoms lay on a severity scale was also mentioned by several respondents as part of the reported desire for screening, as opposed to a binary “present” or “not present” assessment outcome more akin to a diagnosis:

I think I’d be more comfortable with saying like, you’re showing depressive symptoms of moderate severity. Here’s a list of the conditions that typically show these symptoms.

Transcript 16

Interviewees also expressed the importance of evidence-based assessment in encouraging trust in both the assessment itself and the results:

So yeah, I think it’s helpful if it would, if it was like a regulated test and things like that.

Transcript 4

The importance of an evidence-based assessment was also highlighted by interviewees if the results of the assessment were intended to be shared with the user’s health care provider (eg, GP, psychiatrist):

That’s [sending results directly to a health care professional after a digital MH assessment] a really good idea. If the app had been developed by professionals and it had been approved as being scientific, meeting all the criteria.

Transcript 15

One source of apprehension disclosed by interviewees regarding engaging with mental health apps is the potential for nefarious digital tools (eg, apps, websites). This included tools that were either making recommendations not based on clinical evidence or tools that were perceived to have been designed with the sole intention of making a profit for the developer, rather than to help app users:

There’s got to be something like that out there, you know, like dodgy websites, which are not quite breaking the law, but they’re not following the proper recommended guidance. Some of them can look really professional but the real interest is money really.

Transcript 15

I don’t like the idea of people profiting off of people’s data about mental health.

Transcript 2

A concern shared by interviewees was the lack of regulation and quality assurance:

I don’t know who would monitor or approve or disapprove of these apps, because there’s so many thousands, tens of thousands of apps for everything and there’s no sort of overseeing body which could say “you can’t say that.”

Transcript 15

Some interviewees expressed concerns about the appropriateness of the self-help tips offered in mental health apps. This concern focused on self-help advice offered in mental health apps often being designed for general well-being, and thus, typically not being beneficial for individuals experiencing symptoms of a mental health disorder:

[Mental health apps] try to provide solutions, and they say that “oh, just do a breathing exercise.” And you know, I used to be a yoga instructor. So I know a lot about breathing and breathing exercises, and they’re really good, but if you’re if you’re struggling, if you know if you’re having a panic attack, breathing is not going to help.

Transcript 2

Concerning the minor theme of background and development, there was interest in ensuring that key stakeholders were included in the development and design of mental health assessment apps. In terms of including clinicians in the design, it was reported as important to verify that mental health assessment apps are designed to best support existing care pathways and address the waiting lists to access mental health services:

I guess I’d [be] keen to, yeah, to want mental health professionals to have sort of contributed to the app to make sure it is going to reduce waiting lists, rather than just do nothing.

Transcript 16

Beyond designing an app that can offer benefits within the clinical pathway, there was a mention of the importance of engaging with the intended population during development to establish which features and designs are required for accessibility:

So I guess in the design phases, checking with those individuals with those conditions and sort of saying, is this accessible to you? What would make it accessible to you?

Transcript 16

Perceived value is a potential facilitator of app uptake. That is, the benefits conferred by demonstrating the efficacy of the mental health app, especially when the app offers therapeutic or self-help functionalities:

I think it always helps to know that it’s been effective for people like[...] especially for doing like therapy skills and therapy techniques.

Transcript 7

However, the caliber of the evidence required to demonstrate the app’s efficacy was not disclosed, so it is unclear whether positive reviews or word-of-mouth recommendations are sufficient or if a more formal evidence base is necessary to promote engagement with mental health apps.

The perceived value of the app as determined by the user was deemed to impact app use and linked to drop out. Interviewees stated that they had forgotten to use or lost motivation to use apps if they did not find them valuable:

But yeah, I didn’t really find much value in it and I, I forgot to use it [...]. So I just kind of in the end, I just didn’t, didn’t bother.

Transcript 11

With interviewees additionally stating that if the novelty of the app wears off over time there is a risk of dropout:

I only really used that for a few days. It’s one of those things, you know, download something and it looks really cool and then the novelty wears on really fast

Transcript 5

In contrast, interviewees discussed how apps perceived as fun encouraged continued use by incentivizing users to return through gamification:

there's sort of incentive to play every day and you can buy him [habit tracker app mascot] outfits and stuff like that, and it's just a bit more fun

Transcript 4

#### Attitudes

Attitudes toward using digital technologies for mental health were revealed to be an important theme, with issues surrounding (1) openness to digitalization, (2) interest in using a digital mental health assessment, (3) apprehension, (4) the value of reputability, and (5) stigma emerging as key minor themes.

In this study, attitudes regarding being open to digitalization were overwhelmingly positive:

Yeah, I’m more than happy with that don’t have an issue at all. I think we use apps all the time, don’t we for pretty much everything.

Transcript 11

In contrast, the importance of being interested in using a mental health app in the first place was highlighted:

[...] don’t want to [use a mental health app], I’m not interested. I’ve never thought about any of them. and not being interested in using a mental health app when symptoms are not present

Transcript 6

I’m not exactly sure why but have a huge desire not to monitor anything when, when I’m feeling fine.

Transcript 6

Apprehension was revealed to be an important factor in determining mental health app use, particularly regarding the lack of face-to-face contact:

I find the lack of human contact, I find it, I find it the downside of it.

Transcript 14

and distrust for apps that appear in advertisements:

I’m suspicious of some of the things that I see pushed in ads and things like that.

Transcript 4

In contrast, regarding the minor theme of the value of reputability, interviewees stated that app endorsement from a source that the individual perceives as reputable, such as a trusted institution (ie, a university, health care service, such as the National Health Service, a mental health charity) or a direct recommendation (ie, from a health care provider, or a trusted friend or family member) could drive their intention to use the app:

I suppose anything reputable. [...] any institution that I feel comfortable with, be it a service or a, or research department, that would influence me? Yeah.

Transcript 6

If it was given under an accredited body like NHS Wales, and Mind, then that will put a slightly different complexion on it for me.

Transcript 3

But also friends and family. If they’ve used it, that’s important.

Transcript 11

Finally, stigma was seen as an important driver of app use, with interviewees expressing that a mental health app could reduce feelings of judgment and isolation:

I don’t know if that maybe helps with the stigma a little bit. If you know that all of your friends have got that app downloaded, then you know that you’re not the only one that’s really struggling.

Transcript 7

#### Safety

A major theme identified in this study was safety, comprising four minor themes: (1) data security; (2) disclaimers; (3) concerns about the potentially triggering nature of digitally delivered mental health assessment results; and (4) data sharing, consent, and safeguarding.

Interviewees expressed the importance of data security of mental health apps:

Well, all I can say is obviously privacy, confidentiality is extremely important.

Transcript 6

I can see that the GDPR and privacy concerns would be high.

Transcript 1

The level of trust interviewees expressed regarding apps’ data security policies varied, with some stating that mental health apps are unable to provide basic data security:

At the moment, don’t quite think we’re here. Not quite there. Purely and simply, from my perspective, in and around the areas of control and security. That’s my own perspective. I don’t think we’re ever going to be 100% secure.

Transcript 12

Interviewees who expressed this sentiment also mentioned concerns about their data being easily available to those with nefarious intentions, such as hackers.

[...]it seems self-evident that if you’re talking about your own mental health, you don’t want the whole world to be able to hack into it in any way.

Transcript 6

Other interviewees instead assumed the relevant steps had been taken to protect their data:

I guess I’d be sort of confident if this app came into being with the people behind kind of gone through the process of making sure information is kept confidential. I wouldn’t necessarily have particular worries about kind of privacy or safety. I don’t think.

Transcript 16

Some interviewees expressed that their use of technology and previous interactions with nonmental health apps (eg, banking or social media apps) influenced their level of trust in data security on mental health apps. They stated that they were already comfortable providing sensitive data to nonmental health apps, and thus, did not understand why providing data to mental health apps would be any different:

I wouldn’t say that I have any major concerns with regards to privacy or safety, I think because so much of my life is on my phone and it’s kind of you know, there’s so many companies that know so much about you know, if you sign up to things on your phone or apps or whatever.

Transcript 5

In terms of actions, app developers could ease data security concerns, and interviewees mentioned a preference to remain anonymous while using the app. This reduces the potential data security risks related to data leaks:

But in terms of that being traceable, or anything that doesn’t really concern me. I’d just rather not have to put in things like my full name, date of birth card details, that sort of thing.

Transcript 4

Interviewees expressed that the inclusion of a diagnostic disclaimer was important to ensure that app users were aware that the mental health assessment results were intended solely for screening rather than for diagnostic purposes. Interviewees additionally stated the importance of a mental health assessment app disclaimer communicating to users that any results should be shared with a relevant clinician for further confirmatory evaluations:

I think the app was using it gave you different score[...] 21 to 30 it seems like you’re likely to have ADHD, but you need to go to speak to a professional bringing [screen]shots of this app or take a print out and say perhaps the fourth one was you’ve definitely got ADHD from what you’ve told us, but you still need to be assessed by a professional. So it was like a really gentle thing.

Transcript 15

Interviewees verbalized concerns over the potential triggering nature of receiving mental health assessment results digitally, using the words “frightened,” “worried,” and “overwhelmed.” There were 2 dimensions related to this issue, namely, concerns about the assessment’s diagnostic accuracy:

[...]if an app tried to do diagnosis, and it completely got everything wrong, then it could lead to the person getting a bit too overwhelmed.

Transcript 13

and a potential lack of timely access to clinicians with whom one could discuss and validate the results:

And I can’t see why anybody else would not find it frightening because if somebody was going to use this method, then let’s just you know, you can’t get a doctor’s appointment, a physical appointment about three weeks down here. So I would have to sit on that and then get a doctor’s appointment. So I, I would find it, and also seeing it in black and white what was wrong with me. No, I think that would send me into a spiral.

Transcript 3

In contrast, another interviewee expressed that receiving the results of a mental health assessment app may be reassuring rather than triggering based on their own previous experience using digital mental health self-assessment tools:

Yes, it [receiving mental health results from a digital tool] wasn’t intimidating if you like. When I got the high score, I thought “Yeah, this is good,” I didn’t see it as a negative thing at all.

Transcript 15

Interviewees praised the benefits of having an app that is integrated into the existing health care pathway where the results of an assessment would be directly sent to a health care professional:

I’d prefer that [mental health assessment results being sent directly to a clinician] rather than letters really. It’s much quicker, instant information.

Transcript 11

However, related to the minor theme of data sharing and consent, interviewees indicated that the integration of app data into the existing care pathway must be authorized by the app user through a consent-driven approach to result sharing:

[...]you shouldn’t be forced into presenting this information [results from a digital mental health assessment to a health care professional].

Transcript 13

with a preference for multiple opportunities to provide consent (eg, at the point of download of the app and at the point of sending the data to a clinician) to share their mental health data with a relevant health care professional:

I would just like to consent at the moment of sending. So I think there’ll [have to] be two ways of consent. There is a general consent when I joined the app, right? But there is also a consent before it’s actually, actually shared. Yeah, if there are these two layers of consent.

Transcript 13

A preference for risk safeguarding was expressed with the suggestion that mental health apps should be built with functionality to override the consent requirement if the user discloses a substantial level of risk:

I also think it should be a bit of a disclaimer like if you disclose a level of risk, we will send it on automatically to a mental health care professional just so people who are disclosing levels, like levels of risk aren’t[...] just left to it.

Transcript 16

as well as including crisis signposting in app:

...when you are logging your mood, if you log you know, really bad, it might prompt you to say, you know, I noticed that you’re not feeling so good, would you like to call this number or would you like to look at this website.

Transcript 4

In addition, there was a suggestion to pair requesting consent to share mental health data with health care clinicians to facilitate informed decision-making, wherein the app user has the agency to decide which care pathway they prefer to receive help from:

[...] you could have [an] option saying “okay, we’ve identified this, where would you, we recommend that you should go to a GP, you know, but we also have these, these options. Where do you consent for us to send your information?

Transcript 11

#### Impact

The impact of a mental health assessment app on mental health care provision was deemed an important theme in this study, with interviewees discussing issues about (1) the effects on the existing care pathway and (2) support and signposting outside formal health care. Regarding the former, interviewees positively viewed the impact that mental health assessment apps could have on existing care pathways:

So to actually have that [results of a mental health assessment completed in an app] sort of sent to someone [a clinician] and have that resource available, I think that’d be really, really valuable, actually.

Transcript 5

I have [had] GPs in the past that haven’t really had much training or like knowledge in mental health. So I think maybe if I could then go to my GP and say, “Well, I’ve done this and it’s said that it indicates this diagnosis” I think it could maybe like make that easier.

Transcript 7

However, the importance of the role of the health care professional in reviewing any output from a mental health assessment app:

I think it’s really important to pass on this information for the counselor of GP or whatever to look over and then they’ll decide whether that information is accurate

Transcript 13

It means the human being can just kind of clarify answers and make sure that whatever decision the app has come to is actually accurate [unintelligible] just checking up with the actual person.

Transcript 16

It should be emphasized that any app integrated into existing care pathways should support clinicians and not replace them:

It’s about accelerating my access to a person or a group of people that can help me and not replacing that with a digital application because I don’t believe in that.

Transcript 2

Interviewees expressed that completing a digital mental health assessment could facilitate discussions with their health care providers:

One of my big worries is booking doctor’s appointments and struggling to word what I am trying to put across. I think a report sent across with an opinion or something, yeah, to my GP so that I do not have to do it myself and it sounds more reliable, I suppose, would be really, really helpful actually personally.

Transcript 4

and could help triage patients to appropriate health care professionals or services:

I think on the whole, the app could make an accurate assessment of yeah, which pathway was best.

Transcript 16

[...] for lower risk perhaps you can still defer, defer them to somewhere else to see if you can offload the pressure load of the GPs and leave it for for higher needs patients, I guess.

Transcript 14

as well as offering tracking of mental health symptoms during treatment, which can be shared with clinicians to inform:

I can show that [data from mental health tracking app] to her [community psychiatric nurse], and she can take a screenshot for my records, and then we can just discuss if there’s any external triggers or you know how I feel about noticing the slow change in my mood by going back to the app seeing the graphs and the change.

Transcript 15

In contrast, interviewees expressed concerns regarding the potential of a mental health assessment app to further overwhelm existing services, creating a bottleneck where clinical needs are revealed, which cannot be met by existing mental health services:

[...] is this going to open the floodgates and then there aren’t the services to meet all the huge need that you uncover.

Transcript 6

There are additional concerns that the quality of mental health care may worsen because of digitization, with face-to-face care being replaced with digital care:

Like if it’s [an app] an option that is there as well as, but I would, I would be, my worry will be if that one, one day that[...] would be the substitute to be seen a person

Transcript 14

Even when it is potentially inappropriate for higher-severity or complex mental health symptoms:

I guess my like, that my concern is that sometimes would it just become like, they just start signposting you to the app instead of... even when it is not appropriate.

Transcript 7

With this in mind, interviewees stated that there was a need for clinicians to refer patients to mental health apps to justify digitization:

I think I would ask why the doctor is presenting me with an app. When it’s something we couldn’t do with ourselves

Transcript 3

In terms of the impact a mental health assessment app could have on support and signposting outside of formal health care, interviewees discussed the opportunities that mental health assessment apps can offer in terms of encouraging help-seeking:

I think that would be the sort of ideal outcome that I would look for if I was completing some sort of assessment. Obviously, you’re completing it because you think that there might be an issue. And then, at the end of the day, that's the kind of first port of call that you know, you would go and see a health professional

Transcript 5

I think it can be difficult sometimes to actually go and ask for it. Even if you know you’re really struggling, it can be hard to actually go and have a conversation. So I think you could maybe help people who are quite anxious about going to see their GP if they know that they’ll get some information first.

Transcript 7

In addition, interviewees expressed the potential for support to be provided to users while they were on a waiting list for further assessment or appropriate treatment:

[...] if you’re on a waiting list being told that there are these apps that might be able to help you while you’re on that waiting list to manage, you know, managing emotions or whatever it is, then that would be good.

Transcript 1

In addition to improving access to faster help by signposting support outside of traditional care services:

it might make it [wait times] shorter because if they are then being directed to other quicker options. That might be better for the traditional services.

Transcript 11

as well as the app having the potential to provide users with sources of help and self-help tips, rather than solely focusing on an indication of a diagnosis:

[...] as long as it had additional information and useful links and things like that, that go alongside it, and indicators of where they can go to maybe get help, or you know, to help themselves while they’re waiting to get an appointment, for example, then that would be that would be more useful than just an indication of a diagnosis and nothing else.

Transcript 1

Potentially, like, self-help type resources as well. So like information, and I guess there are sometimes like courses you can do on like anxiety management, or whatever.

Transcript 16

#### Functionality

This major theme included the following minor themes: (1) usability, (2) features, (3) customization, (4) personalization, and (5) interoperability.

In terms of usability, interviewees commented on the importance of app design, with a focus on a preference for a professional interface that may foster a degree of trust in the reputability of the mental health app:

I guess, just the, you know, the app, if the app looked professional, and it looked well done, and you know, it doesn’t look like someone had coded it together in their bedroom or something like that.

Transcript 5

In addition to the importance of app design, interviewees expressed that ease of use facilitates app uptake and continued app use:

I think there should be an app that’s quite basic and as well as functional but easy to understand.

Transcript 13

with perceived difficulties in using the app constituting a potential barrier to disengagement with the mental health app:

I think being user friendly is to the real... is the real key and anything that was too wordy to read on a screen I was just 'Oh well I can’t deal with that.

Transcript 5

As part of usability, interviewees commented on the importance of in-app guidance to support users in their interactions with the app:

I think it would have been better if it was more guided. You know, so perhaps, set a set schedule of tasks to do or something. I am sure there was a lot of information in it, but it just was the format that[...] I didn’t like really.

Transcript 11

Therefore, this could be a feature included in the app to promote ease of use, particularly in self-help apps that often reportedly rely on self-motivation:

I think a lot of them are more like self-help geared so I suppose if you struggle to do sort of stuff on your own, then you might struggle to use the apps, because they are very self-guided.

Transcript 7

Finally, in terms of usability, during the first use of the app after downloading, there was a preference for the ability to explore the app before any sort of commitment was asked (eg, creating an account or having to enter payment information). Part of this preference was related to being able to ensuring that the features included in the app were relevant to the needs of the user and suited their preferences:

I open one and it’s immediately like, sign in, well I might just delete that one because I don’t want to sign in and I’ll go for the next one. Whereas if it’s open up, and I could take a look around the features and then decide, oh, actually, there’s something that I’d like to use, then I’d be happy enough to sign up. Yeah.

Transcript 5

A common feature mentioned by interviewees was tracking functionality, including monitoring of the user’s mood and mental health symptoms:

[...]it would be best if you could sort of track your mood over weeks or months or days. Maybe have graphs or a little bit of information available on[...] what’s average for you.

Transcript 4

In addition, interviewees expressed that an app with functionality to monitor both physical and mental health data would be useful for identifying triggers for changes in mood or mental health symptoms:

It’s all in one which is quite useful because then you can see like... hmm... I think it helps identify triggers sometimes like, yeah, if [I] had a really bad night’s sleep, then that might explain why the next day, I wasn’t feeling very good. It’s quite helpful having it all in one place.

Transcript 7

The final type of tracking that interviewees regarded as helpful was habit tracking, that is, a functionality where a user can set tasks to complete throughout the day with the help of reminders:

[...]you can set tasks every morning like brush your teeth, get out of bed, go outside, then like by ticking all of those off your little bird buddy goes off on an adventure.

Transcript 4

Interviewees expressed that in-app reminders are a helpful feature and, in some cases, are the most useful features:

I think the most useful things for me are setting reminders to take my medication on, on an app.

Transcript 4

Interviewees also highlighted the importance of social connectedness and the value of a mental health app that offers peer support:

And so from my perspective, my personal opinion, is that an app would be a value if it helps me connect with other people.

Transcript 2

In terms of features specific to mental health assessment, interviewees emphasized the importance of comprehensiveness:

definitely more comprehensive assessments that kind of go through different types of diagnosis and criteria, like sounds like would be a lot more very valuable than just putting everyone in like either depression or anxiety buckets

Transcript 10

And having multiple answering modalities beyond only offering predefined answer options:

It depends on how am I answering these questions? I mean, is it a free text? is it voice? is it video? Is it typing? Is it selecting? Because if it’s yeah, if the questions are open and I can be very subjective about it, then I would like that, probably. If it’s just selecting, like PHQ and GAD, that’s kind of, I don’t find value in those.

Transcript 2

With free-text options being important to add nuance:

if you had a multiple choice for you to be for there to be an option at the bottom of it to say, you know, why did you answer this way or is there any additional information that you want to give because often there is

Transcript 10

However, despite the positive remarks provided about the possible features offered within mental health apps, interviewees reported that some features included within apps previously used by interviewees were counterproductive. Interviewees commented that some app features interfered with the usability of the app:

[The app] helps you plan your day and to get less distractions and feel more organized throughout the day. It was beeping and it was saying 'Have you completed the task you set this morning? How do you feel about the task? Did it go well?' It was all these bully questions. [...] It's too intrusive. [...] I just found it really annoying. Sometimes I didn’t want to answer any questions. But if you didn’t, you'd get a prompt an hour later or something.

Transcript 15

Therefore, customization of app features to users’ requirements and preferences (eg, changing reminder frequency, personalizing a crisis plan, changing the design) was also highlighted as an important aspect of functionality to avoid the features becoming irritating or counterproductive, leading to potential discontinuation of use:

I control the level of notifications. You know, some people might like lots, some might not want any. You know, times of notifications, hints, tips, that sort of thing.

Transcript 11

I think having the ability to set notifications at a specific time or just once a day, is really helpful to remind me to actually log what I’ve done and things like that.

Transcript 4

In addition, customization can offer users the freedom to choose their preferred method of engagement with a mental health app. Interviewees expressed interest in various modalities of information delivery within apps, beyond written text. Specifically, there was an interest in apps offering videos that provide information about mental health disorders:

I think having a whole library of videos [for mental health information] would help.

Transcript 13

In addition, interviewees expressed that they would find personalization of a mental health app output (eg, self-help recommendations, psychoeducation) valuable:

Yeah, I think as I mentioned, sort of programmed approach or maybe personalized in some way, depending on maybe an initial assessment so it's you know more individualized.

Transcript 11

Interviewees reported an interest in apps that are interoperable, providing the ability to link mental health apps with other health data collection devices, specifically physical monitoring devices such as Fitbit:

[...]it could link with, you know, Google Fit, you know, because obviously exercise and you know, mental wellbeing do go together. So, it’d be good to just link in with that.

Transcript 11

## Discussion

### Principal Findings

This study aimed to explore the perspectives of individuals with lived experiences of mental health concerns on mental health apps designed for self-assessment and triage. A semistructured interview approach was used, and the findings revealed 6 key themes: availability, accessibility, quality, attitude, safety, impact, and functionality. These themes provide insight into how potential users experiencing mental health symptoms may perceive and use mental health self-assessments and triage apps. These findings can help app developers design and improve these technologies to better support the mental health needs of current and potential future users.

### Availability and Accessibility

Concerns pertaining to the theme of “availability and accessibility” were raised by interviewees in this study. They noted that mental health symptoms can make it difficult for individuals to engage with digital mental health assessments and triage tools. Indeed, mental health symptoms and cognitive deficits associated with conditions such as depression and psychosis may make it challenging for people to use such technologies [36]. This highlights the importance of designing accessible and user-friendly digital mental health tools. To achieve this, app developers should consider involving real-life patient populations in the design and evaluation of new technologies [37] to ensure that the tools are appropriate and effective for intended users.

In addition, the risk of digital exclusion was raised by the interviewees, particularly in relation to older individuals who may not be digitally literate. Previous research has shown that older adults are less likely to use technology and the internet compared with younger individuals [38]. To address this, training in digital tools for mental health may prove effective in supporting older adults to engage with these technologies and avoiding exclusion from accessing important support and resources [39]. Interviewees also mentioned that it was important to ensure that digital tools were not the only “front door” to care services, guaranteeing that those who may be excluded from engaging with such tools are still able to access help through existing pathways.

Moreover, interviewees raised concerns about the lack of diversification within the app landscape, in terms of which mental health disorder apps were available. Specifically, many interviewees referred to the perceived lack of available apps designed for neurodevelopmental disorders. To address this, app developers should avoid contributing to the perceived oversaturation of apps for general common mental health conditions (ie, depression and anxiety disorders) and instead identify opportunities to design apps for a more diverse range of conditions, especially as this study demonstrates a clear interest in accessing such apps if they are available. In addition, investigating gaps in the app market and investing in co-designing approaches with users could help improve uptake among typically excluded populations [40,41].

Finally, interviewees suggested that offering mental health apps as an employee benefit can improve accessibility. Previous research has shown that digital interventions for employees experiencing psychological distress can be highly scalable and cost-effective [42-44] conferring positive impacts on well-being and productivity outcomes such as sleep, stress, and presenteeism [44].

### Quality

Interviewees identified the “quality” of available digital mental health assessments as an important consideration. They mentioned that the subjective nature of mental health symptoms could make it difficult to accurately assess and diagnose mental health conditions. However, it is worth noting that this has also been mentioned in traditional face-to-face mental health assessments. The risks of inaccurate self-reporting due to recall and perceptual biases are widely recognized [45]. For example, previous work has demonstrated only a moderate correlation between self-reported length of sleep and actigraphy measurements, with individuals overestimating sleep duration [46]. In addition, prior research on bipolar disorder shows that individuals have trouble remembering the details of earlier manic or hypomanic episodes [47,48]. This subjectivity and difficulty in accurate reporting can lead to the misclassification of symptoms and, in turn, misdiagnosis.

Furthermore, interviewees commented on their concerns that the available digital tools designed for mental health self-assessment could provide inaccurate diagnostic results. Indeed, many of the currently available apps designed for mental health do not provide evidence of their efficacy, accuracy, or effectiveness [49,50]. There is limited high-quality evidence on the diagnostic accuracy of available digital mental health assessments, which vary in their performance when compared with a gold standard clinical interview, with some showing poor discriminatory and differential diagnostic performance and others demonstrating excellent accuracy [51]. To address this, interviewees emphasized the importance of ensuring that digital mental health assessments are clinically validated and evidence-based, particularly if the results are intended to be shared with health care professionals. A further way to address this concern is for app developers to create apps that offer screening and an indication of where a user’s symptoms fall on a severity scale rather than a diagnosis, particularly given that interviewees in this study expressed a preference for screening overdiagnosis. To increase engagement and build trust in the app and its assessment results, there should be functionality to easily share results with a health care professional for evaluation and confirmation.

Another concern raised by the interviewees was the potential for nefarious digital tools and inappropriate information or self-help advice targeting vulnerable individuals. Previous work has demonstrated that these concerns may be well founded, as some available mental health apps inappropriately promote the medicalization of normal mental states [52]. These concerns also reflect the current lack of regulation and quality assurance in the digital mental health field [53].

### Attitudes

The theme of “attitudes” toward digital mental health technologies was also identified in the study as an important factor influencing the use of mental health assessment and triage apps. Similar to previous research, openness to digital technologies was identified as a key driver of engagement in digital mental health technologies [54]. In addition, and perhaps unsurprisingly, this study demonstrated the importance of interest in using these technologies, with a lack of interest constituting a significant barrier to the initial uptake. In fact, some interviewees described lack of interest as the fundamental reason for not currently engaging or not intending to engage with mental health apps. Previous research has shown that providing relevant and customizable content can increase interest, and in turn, the uptake of digital mental health technologies [55]. Therefore, engaging with stakeholders in the design phase of such technologies is critical to ensure that the content is relevant to the intended user population, driving interest, and engagement. However, it is important to note that in some groups, their lack of overall interest in such digital tools will always remain a barrier, and, as previously stated, nondigital access to services must be maintained so as not to inadvertently exclude individuals.

In addition, the value of reputation was identified in this study, supporting previous studies [55]. Interviewees emphasized the importance of the app being endorsed by a reputable source, such as a research institution, health care provider, trusted friend, or family member. This can help increase trust in the app and reduce stigma. Interviewees also expressed distrust of mental health apps that are advertised in paid advertisements, as advertising may indicate that the app was designed primarily for commercial gain rather than therapeutic benefits. Therefore, the initial engagement and use of an app can potentially be increased by improving the prospective user’s perception of the app’s reputation through endorsements from trusted individuals or organizations and by ensuring that any paid advertisements are not perceived negatively.

Furthermore, beyond just passive reputation, this study additionally determined that an active recommendation of a specific app by one’s health care provider is an excellent strategy to encourage uptake, as this fosters a sense that the app will be both highly relevant and effective in managing the patient’s conditions and needs. This reflects previous qualitative findings regarding the potential importance of a health care professional’s recommendation to encourage interest in and uptake of mobile apps, specifically for managing depressive symptoms in primary care [56]. Given this evidence demonstrating the potential influence of health care professionals on the uptake of digital mental health tools, ranging from assessment and triage to management (ie, symptom tracking and self-care as an adjunct to formal pharmacological or psychological treatment), primary care clinicians should be aware of their capacity to signpost patients and identify appropriate opportunities to do so. There are resources available to support clinicians in identifying high-quality apps to recommend to patients, such as the Orcha app library [57] and the American Psychiatric Association’s app evaluation model screener [58], which could be valuable assets in encouraging digital tool uptake in patients.

### Safety

With respect to the “safety” theme, some interviewees raised concerns regarding trust in digital mental health technologies, particularly in terms of data sharing and anonymity. Some individuals may be wary of sharing their sensitive, personal information or seeking support through digital mental health technologies because of doubts regarding the confidentiality or anonymity provided by these apps. Unfortunately, these concerns may be well founded, as despite mental health apps collecting some of the most sensitive personal information, their data security provisions often do not differ from those of nonmental health apps [59]. Interviewees expressed that to address these concerns, digital mental health apps must provide clear and transparent information on how they handle user data. Lamentably, despite interviewees conveying a desire for this information, many mental health apps do not offer a privacy policy to users [60]. Of those that do provide a privacy policy, many demonstrate low readability scores [61], potentially fostering a sense of mistrust in how collected data are being analyzed and used. Conversely, some interviewees expressed a more nonchalant attitude regarding data security.

Interestingly, this study identified how one’s interaction with nonmental health apps can influence one’s perception of the data security of mental health apps, with interviewees claiming that they have minimal concerns about sharing sensitive data as nonmental health apps, specifically social media and banking apps, already collect data of a perceived similar sensitivity. Despite this apparent lack of concern on the part of app users, app developers are still responsible for upholding the required levels of data security.

In addition, interviewees communicated the importance of providing users with the option to opt out of data sharing. Interviewees recommended that, ideally, this opting-in would include asking for explicit consent at multiple time points during the use of the app, before any data were inputted, and then again before sharing any calculated results with a care professional. Currently, many publicly available apps treat continued use as a proxy for the user’s consent [61] to collect and analyze mental health data rather than asking explicitly for consent periodically, despite potential changes to the app’s data collection and analytic strategy, data sharing, or privacy policy. In addition, even if mental health apps were to adopt a consent-driven approach (ie, where consent is obtained at multiple time points), problems related to consent would persist because of the low readability scores of app privacy policies. As discussed above, users may not truly understand what they are consenting to [61]. However, interviewees expressed that, in some cases, it may be necessary to override the user’s consent to share results with a health care professional if they disclose a substantial level of self-harm or suicide risk.

Another requirement raised by the interviewees was the need for a diagnostic disclaimer. This means that nondiagnostic mental health apps should clearly state that their assessments are intended for screening purposes only and should not be used as a substitute for a professional diagnosis. Many available mental health apps lack such disclaimers [62]. Providing such a disclaimer clearly in an app store description can help prevent users from overreliance on the results of these assessments and may encourage them to seek confirmatory assessments or support from qualified professionals.

### Impact

The theme of “impact” was also identified in the study, with interviewees commenting on the effects that digital mental health assessments can have on current care pathways. They suggested that digital mental health assessments can facilitate informed discussions with health care professionals, echoing a sentiment identified previously in the user feedback of a novel digital mental health assessment [63].

However, some interviewees also expressed concerns that mental health apps could further overwhelm existing mental health services, particularly if they led to an influx of new users seeking support. One method proposed by interviewees to proactively avoid overwhelming services was to co-design apps intended to be implemented in existing clinical pathways with relevant care professionals. This would ensure that they are a valuable tool and not a hindrance to the established delivery of care [64]. Although digital mental health technologies will not immediately solve issues of long waiting lists and a lack of trained mental health care professionals, they can allow cost-effective and time-efficient collection of patient and symptom data. In addition, interviewees expressed concerns that the digitization of existing mental health services may result in poorer care, particularly for those with more severe or complex mental health symptoms. This view supports the notion that digital tools should be used for augmentation, rather than replacement, of existing services to support clinicians in the delivery of care.

A concept that was discussed positively by interviewees in this study was the potential of apps to support the waitlist management of mental health services. Mental health triage apps have the potential to direct patients to the most appropriate formal care pathway or other services (ie, local charity, self-help resources, psychoeducation) based on their symptom profile, potentially alleviating the burden on the health care system by providing individuals with mild or subthreshold symptoms with self-help tips and psychoeducation, reserving GPs, or specialized services for more severe or complex cases. Moreover, interviewees overwhelmingly expressed an interest in mental health apps that can support users while they are on the waiting list before receiving formal treatment by offering not only an assessment and triage, but also self-help tips, psychoeducation, and sources of help. Psychoeducation has been demonstrated to increase mental health literacy, decrease feelings of stigma, and increase intention to seek help [65]. In addition, psychoeducation has been shown to improve outcomes in bipolar disorder, as measured by hospital admissions [66]. Considering the wait time between assessment and psychological treatment in the United Kingdom and the associated potential for the deterioration of symptoms and well-being [4,5], any opportunity to arm individuals with resources to support their own mental health should be explored. However, beyond the potential to improve patient experience while on the waitlist, more work should be done to investigate the therapeutic benefits of providing self-help information to patients awaiting formal treatment.

### Functionality

Finally, in terms of “functionality,” similarly to previous work, interviewees mentioned that ease of use and in-app guidance would increase app use [55], especially for apps that require a high level of motivation, such as mental health apps.

In terms of assessment specific features, interviewees mentioned the importance of comprehensive assessments to ensure that the complete picture of mental health symptoms and contributing factors is captured. In addition, interviewees highlighted the importance of including varied answering modalities to capture the nuances of experience associated with specific symptoms. This reflects the sentiment from user feedback of a digital mental health assessment, which found that questions with predefined answer options cannot always correctly capture symptoms with requests for the ability to add free-text data [63]. This study demonstrated that users are keen to engage with apps that offer a wide range of complementary functions beyond self-assessment to support mental well-being. The desirable app functions mentioned by the interviewees included mental health symptoms and habit tracking. Mental health symptoms and mood tracking are popular features of mental health apps [29] and can be valuable to users who wish to increase their awareness of their mental health concerns [67]. Beyond only mental health symptom tracking, interviewees expressed a preference for apps with the ability to track physical health alongside mental health in a single app, thereby providing more comprehensive insights (ie, menstrual cycle, exercise). Further demonstrating the importance of ease of use from the perspective of interviewees in this study, apps that offered interoperability and facilitated the ability to link mental health apps to other health data collection devices (eg, Fitbit) were preferred. This data sharing between devices could combine physical health data (ie, heart rate, sleep amount, and quality) with manually entered mental health symptom data to help individuals identify mood triggers and patterns as well as self-management of their mental health symptoms.

In addition, the opportunity to gain peer support through mental health apps was considered important by interviewees as it has the potential to address the missing “human” aspect of digital mental health technologies. In addition, some interviewees mentioned that in the past, using social media for peer support enabled them to learn more about their illness from other individuals who had similar symptoms, which is why they thought it would be beneficial to have a mental health app. Peer support has been shown to improve feelings of hope, empowerment, and social functioning [68], with the consolidation of early stage evidence investigating digital peer support demonstrating acceptability and positive effects on functioning and outcomes [69].

Although reminders were viewed as an important and useful feature of mental health apps, interviewees stated that too many reminders and notifications were also considered intrusive and could lead to app discontinuation. However, facilitating the customization of these features could promote engagement, further corroborating previous findings [63]. In addition, offering different modalities (eg, text, audio, and video) is seen as an important factor in engagement and app usage. Therefore, app developers should ensure that such features are easily customizable to optimize usefulness from the user’s perspective (ie, by ensuring that reminders to use the app are delivered when the user can do so) and are not overwhelming.

As discussed above, there was interest from interviewees in resources to support self-management of mental health symptoms while on a waiting list (ie, self-help tips and psychoeducation). However, it is important to note that personalization was expressed as important to interviewees, with a preference for personalized rather than generic self-help tips and psychoeducational information. Therefore, although there may be temptation from app developers to create apps using generic self-help, this may impede interest and eventual engagement with their app. Therefore, offering self-help tailored to specific conditions or populations is preferable. Doing so may also go some way toward alleviating some of the concerns expressed by interviewees in the quality theme concerning the appropriateness of self-help offered in apps, providing app users an opportunity to more confidently choose apps based on what is most relevant to them, as well as ensuring that the information is suitable for their mental health symptoms beyond general mental well-being.

Creating an account by entering personal or payment information could be a barrier to app use identified in this study due to a sense of suspicion in users about data mining, reflecting previous work [29]. Interviewees expressed an interest in being able to access the app initially without creating an account, wherein they could ascertain the relevance of the app to their individual needs and preferences before choosing to provide personal details and payment information where applicable.

### Implications for Practice

Apps adopted for mental health assessment and monitoring present an interesting use case of mobile health technology, and our understanding of their acceptability and perception of benefits and barriers to adoption continues to evolve. Findings from these interviews generated valuable insights into putative users’ perspectives, concerns, and preferences regarding the use and design of apps for mental health assessment. Taken together, these results demonstrate that there is real interest in tools designed for mental health self-assessment and triage, particularly when:

1. The self-assessment and triage tool is offered with additional complementary app features that are personalized or can be customized to the individual user.
2. Self-assessment is comprehensive and includes answering modalities beyond selecting predefined answer options to capture the nuances of symptoms and experiences.
3. The tool is easily accessible, as it does not require the entry of sensitive or financial information for use or is offered via a workplace well-being scheme.
4. The tool is recommended by or associated with a reputable source, either passively (ie, developed with expert academics) or actively (ie, a direct recommendation from a friend or trusted health care professional).

However, despite this interest, individuals have real concerns that may impede their uptake and prolong engagement. This study also elucidates some simple ways in which these concerns can be assured:

1. It appears that by engaging in clear, transparent, and accessible communication about the app’s evidence base and privacy policy in the app description, some of the concerns related to “quality” and “safety” can be addressed before the user even downloads the app.
2. Apps that collect sensitive mental health data and have the functionality to share such data with an individual’s care provider should ask for consent multiple times to establish permission to collect, analyze, and then share.
3. Engaging in co-design activities with both users and clinicians can ensure that the app is (1) widely accessible to many users regardless of the severity or complexity of their mental health symptoms, their level of digital literacy, or the specific condition, and (2) can be effectively integrated into care pathways to support care professionals in the delivery of care. Ensuring access to publicly available digital mental health assessment and triage tools can be achieved through a diverse app marketplace offering that includes typically overlooked conditions such as neurodevelopmental disorders.

Technology integration into mental health care presents opportunities and challenges rooted in the intersection of technical and human factors. Building trust with key stakeholders, such as clinicians, patients, and commissioners, on whom the success of digital integration into existing services will depend, is crucial [70]. Careful design, evaluation of tool performance, and establishing relevance to the population of interest [71] are essential to harness a technology’s potential for improving mental health care delivery and minimizing the possible integration challenges arising from the interaction between technical and human factors. In this study, interviewees seemed to positively view the role that digital tools designed for mental health self-assessment and triage could play in established care pathways *if* responsibly co-designed with health care professionals for successful integration to not overwhelm services. Indeed, technology can sometimes create a barrier to forming a strong therapeutic alliance between patients and health care professionals. In this regard, efforts should be made to ensure that technology augments rather than replaces human interactions. A crucial aspect of this will be interdisciplinary collaboration and co-design of tools and integration strategies. In addition, although the potential for service digitization is certainly attractive, it should be approached cautiously. Interviewees in this study reported concerns regarding lower quality of care resulting from the integration of digital delivery, and continuous monitoring and appraisal of care quality are vital following the deployment of digital tools. In addition, engaging in conversations to explain the rationale and benefits of using digital tools in the delivery of digital care will likely build trust and drive uptake in its use.

### Strengths and Limitations

This study has several strengths. For instance, the use of a qualitative approach allows a nuanced and in-depth exploration of a range of potential users’ perspectives on mental health apps specifically designed for self-assessment and triage. Qualitative research is a powerful tool for generating insights and informing practical solutions and real-world actions. By emphasizing participant perspectives, qualitative research can capture thoughts, feelings, and context, pinpointing subtleties that quantitative methods may miss. In addition, the use of PPI in this study allowed for optimization of the study design and materials, including the suitability and relevance of the interview questions to the population of interest.

Despite these strengths, the results of this study should be viewed through the lens of the questions asked in the semistructured interview as they may have shaped the findings. For example, as we specifically asked interviewees about their views regarding the privacy and security of digital tools, it may have overrepresented the importance of this theme when some individuals would not have mentioned it if not prompted.

Furthermore, insights from this study were drawn from a small cohort of participants and therefore cannot be generalized more broadly, particularly as the entirety of the sample was White. In addition, these exploratory interviews provide insights from a population recruited through web-based social media, mainly through Facebook advertisements. Thus, the resulting cohort may be biased toward individuals who are familiar with digital technologies; thus, the findings may indicate a more positive perspective than that observed in a formal health care setting. However, the cohort in this study offered a wide range of perspectives, including individuals without previous experience interacting with mental health apps and services.

### Conclusions

The adoption of mental health assessment and triage apps presents a significant use case for mobile health technologies. Insights from this study indicate user preferences for mental health apps with personalized features, easy accessibility, and that are recommended by or associated with institutions or individuals perceived as reputable. Concerns about quality, safety, and data privacy can be addressed through clear communication, consent-driven data collection and sharing processes, and co-design with users and clinicians. The positive perception of digital tools in established care pathways highlights potential opportunities for commissioning mental health care services and waiting-list management. However, further research is needed to assess the suitability of digital assessment and triage tools for different psychiatric populations, and to determine their impact on clinical outcomes. In addition, steps must be taken to ensure that concerns regarding the potentially detrimental impact of digitization on care quality are addressed when referring or signposting to digital mental health tools.

## Appendix

Multimedia Appendix 1 COREQ (Consolidated Criteria for Reporting Qualitative Studies) checklist.

Multimedia Appendix 2 Semistructured interview guide.

Multimedia Appendix 3 Codebook organized by associated themes.

## Abbreviations

COREQ: Consolidated Criteria for Reporting Qualitative Studies
GP: general practitioner

## Data Availability

The data sets generated during and analyzed during this study are available from the corresponding author upon reasonable request.
