# Supplementary material for: Perception of Apps for Mental Health Assessment With Recommendations for Future Design: United Kingdom Semistructured Interview Study
Source: JMIR Form Res. 2024 Feb 23;8:e48881. doi: 10.2196/48881 (PMC10924263; doi:10.2196/48881)
Supplement: Multimedia Appendix 2 [file formative_v8i1e48881_app2.docx]

**Semi-structured interview guide**

**INTRODUCTION**

Interviewer and moderator introduce themselves stating their roles and affiliations.

Interviewer asks the participant to introduce themselves briefly.

Interviewer: Thank you for agreeing to take part in this interview. We appreciate your participation. We are conducting this interview to understand your opinions and/or experience of using digital technologies such as mobile apps for mental health. We need your input and want you to share your honest and open thoughts with us so that we can gather information to help develop an app to assess mental health symptoms.

We have a few guidelines and rules to facilitate our conversation:

1. We want you to do the talking.
2. There are no right or wrong answers. Your experiences and opinions are important to us.
3. The discussion will last for about 30 minutes. Please silence your mobile phone if you can.
4. As you know from the information sheet and consent forms you have read and completed before the session, we will record the audio session as we want to capture everything you have to say. The audio recordings will be used to analyse what has been said. You may turn your camera on or off at any point during the session.
5. All your data will be anonymised (that means your name will be removed) and personal details will be kept in a locked file or on a secure computer only accessible by the researchers. When we present data in scientific papers or conferences only anonymised data will be used.
6. If at any point during the interview you wish to stop and withdraw from this study, please let us know immediately and we will terminate the session. Any interview data recorded up to that point will be removed from our database and will not be included in any of the study analyses if you wish so. You can also decide to withdraw after completing the interview by emailing us within a week from today.

Are there any questions?

Are you happy to begin the interview?

Let’s get started…

**QUESTIONS**

1. Have you ever used an application (also known as an app) for your mental health or wellness?
   - Probes if yes
     - Ask what type of app (e.g., screening/monitoring, appointment/medication reminders)
     - What was your experience like using the app?
   - Probes if no
     - Do you use apps for other health purposes (e.g. fitness, heart rate, sleep)?
     - Are there any reasons why you don’t use your phone for your mental health or wellness?
     - Do you use other digital tools for your mental health such as browsing the internet for websites/smart watches?
     - Have you ever looked up mental health apps in the app stores?
     - If not, what has stopped you from looking for mental health apps?
2. What is your perception of available mental health apps?
   - Probes:
     - What attributes would indicate that an app is genuine and of value?
     - Would recommendation by a healthcare professional/the NHS influence your view?
     - Is there anything that would positively change your perception of mental health apps? (e.g., if it was suggested by a health care professional, if a friend referred you, if it showed you what you would get from the app such as benefits and results)
3. What are your thoughts on using an app to answer questions about your mental health?
   - Probes if feels positive about apps
     - Do you think an app could work as a way of assessing mental health?
     - How many questions would you be willing to answer? How long do you think the assessment should take?
     - How many times a day/week/month would you be willing to answer the questions? Or would you prefer a one-off assessment?
     - We know this may work for you, but can you think of a context in which this may not work?
   - Probes if feels negative about apps
     - Do you think an app could work as a way of assessing mental health?
     - Would you prefer a paper based format? Or in-person?
     - We know this may not work for you, but can you think of a context in which this may work?
4. What are your thoughts on receiving an indication of a mental health diagnosis or its severity via the app or via email (in a digital format)?
   - Probes if feels positive about the app
     - What might be the benefits/risks of knowing one’s own symptom severity or possible diagnosis?
   - Probes if feels negative about the app
     - What might be the benefits/risks of knowing one’s own symptom severity or possible diagnosis?
5. What are your thoughts on the information a person gives on a mental health app being sent to a health care professional (e.g., a GP or a psychiatrist) for them to have a look over and review. An appointment could then be schedules to discuss this information.
   - Probes
     - What do you think might be the benefits of healthcare professionals getting this information?
     - What concerns would you have about healthcare professionals getting this information?
     - Would you prefer that the healthcare professional automatically receives this information or for you to take the information directly to them yourself?

*We are over half-way through the interview, are you happy to continue?*

1. Not everyone is able to access the mental health support that they need and there may be waiting lists to receive support. Do you think that apps could help improve access to help?
   - Probe
     - why/why not?
     - Discussion point: It is important to note that this will not increase the number of healthcare professionals/does not solve the issues associated with the shortage of healthcare professionals in the UK. It will also not guarantee that people will be seen by a healthcare professional in a timely manner.
2. What is your view on the privacy or safety aspects related to support offered through mental health apps?
   - Probe
     - Can you tell me a bit more about why you hold this view?
     - Which experiences or information helped you shape your views on this topic?
   - Probe if they have never tried a mental health app
     - If they use the internet for their mental health, do they feel comfortable doing so?
     - If they use other non-mental health apps, do they have concerns about data privacy and sharing on those apps?
3. What would your ideal mental health app help you do? Can you think of anything you would like to see in future mental health support offered online or through apps?
   - Probe
     - Imagine you were creating a mobile phone app or website for people experiencing mental health symptoms what sort of things would you like to see included that might be helpful? What kind of information, if any, would you expect to see on there?
   - Probe if negative about apps in general
     - Is there any way in which you think digital technologies could improve mental health care?
     - What is the main unmet need currently in mental healthcare and how do you think it could be addressed?
4. Is there anything else that you would like to tell me that we haven’t discussed but you think might be relevant when thinking about mental health and digital technologies (such as apps)?

**DEBRIEF**

We are getting towards the end of this session, thank you for all your input answering the questions!

To wrap it all up, we’ll tell you a bit more about our project and rationale.

We believe there exists a significant unmet clinical need in the early diagnosis of mental health disorders in the UK, with mental health disorders commonly remaining underrecognized. This may be due to a variety of reasons, including a lack of adequate professional training regarding mental health symptoms, time constraints, and a pressure to prioritise physical over mental health.

The COVID-19 pandemic has caused additional psychological distress, with many individuals being unable to access adequate mental healthcare support.

A way to overcome these issues may be to implement a digital tool (such as a mobile app) that has the potential to screen and monitor mental health symptoms.

The information gathered in today’s interview will help us gain a better understanding of the degree of acceptability of using apps for mental health. And it will also inform how we think about a future app designed to assess mental health symptoms.

We will be sending you a debrief and the £15 high street voucher by email as a code that can be redeemed online to spend in a variety of shops. Please be aware that the voucher may be regarded as earnings and may affect receipt of state benefits.

If there aren’t any questions we can say good bye now - thank you, we will be in touch via email.
